# An Optimized Audio–Visual Deepfake Detection Framework Using Mamba Vision Transformers with Linear Complexity

**DOI:** 10.3390/s26206406

**Published:** 2026-10-10

**Authors:** Aisha Arain, Bushra Naz, Sania Bhatti, Faisal Alhwikem, Fawwad Hassan Jaskani

**Affiliations:** 1 Department of Software Engineering, Mehran University of Engineering and Technology, Jamshoro 76062, Pakistan; bushra.naz@faculty.muet.edu.pk; 2 Department of Computer Science, College of Computer, Qassim University, Buraydah 51452, Saudi Arabia; alhwikem@gmail.com; 3 Department of Computer Systems Engineering, The Islamia University of Bahawalpur, Bahawalpur 63100, Pakistan; favadhassanjaskani@gmail.com

**Keywords:** deepfake detection, Mamba Vision Transformer, audio–visual fusion, state-space models, multimodal learning, media forensics, real-time detection

## Abstract

The rise of advanced deepfake technologies presents new challenges to media authenticity, making it harder to spot manipulated videos through traditional approaches. Current approaches have a quadratic time complexity $O(n^{2})$, poor cross-dataset generalization with 20 to 35% accuracy degradation, and inadequate audio–visual fusion mechanisms. This paper presents Mamba-AVGuard, a novel audio–visual deepfake detection framework leveraging Mamba Vision Transformers with selective state-space modeling to achieve linear O(n) complexity within the Mamba encoder streams. The novel framework combines a 12-layer visual Mamba encoder for spatial and temporal facial inconsistency analysis with an 8-layer lightweight audio Mamba encoder for prosodic inconsistency analysis, fused via a cross-modal attention block with ±33 ms temporal synchronization. Experiments conducted on two large-scale audio–visual deepfake benchmarks, FakeAVCeleb and AV-Deepfake1M, demonstrate that Mamba-AVGuard outperforms compared methods with 99.92% in-dataset accuracy and 98.47% cross-dataset accuracy on these two benchmarks. The system achieves a processing speed of less than 1.8 s for 30-s videos, with 56% less memory than the best competing transformer-based method, facilitating a throughput of more than 1200 videos per hour for social media verification and forensic workstations.

## 1. Introduction

The high rate of development of generative artificial intelligence has catalyzed an unprecedented explosion of synthetic media, also known as deepfakes, which present existential risks to digital trust, democratic processes, and individual privacy. These sophisticated manipulations leverage deep learning architectures including Generative Adversarial Networks (GANs), autoencoders, and diffusion models to synthesize hyper-realistic face swaps, lip-sync manipulations, and voice cloning that challenge human perceptual capabilities [1].

Modern deepfake detectors mostly use convolutional neural networks (CNNs) and Vision Transformers (ViTs) to detect the signs of manipulation [2]. Nevertheless, these strategies have severe flaws that preclude practical deployment. First, standard transformer architectures impose quadratic computational complexity $O(n^{2})$ with respect to sequence length, resulting in processing times of 8 to 12 s per 30-s video segment [3]. Second, models trained on specific datasets demonstrate severe generalization degradation, with cross-dataset accuracy drops ranging from 20 to 35% when evaluated on unseen manipulation techniques [4,5]. Third, existing multimodal approaches inadequately synchronize audio and visual streams, introducing 15 to 20% performance degradation due to temporal misalignment [6].

More recent studies have examined state-space models (SSMs) as computationally efficient substitutes for attention mechanisms [7]. The Mamba architecture is a selective state-space modeling architecture with linear complexity O(n) that maintains competitive performance on long-sequence modeling tasks [8]. Vision Mamba extends this paradigm to visual processing through bidirectional state-space scanning of image patches [9]. Despite promising results in image classification and video understanding, the application of Mamba architectures to multimodal deepfake detection remains unexplored.

Audio modality also poses extra challenges to detecting deepfakes. Synthetic speech and voice cloning technologies have attained truly impressive levels of fidelity, producing prosodically natural utterances that evade acoustic analysis [10]. Lip-sync deepfakes exploit the temporal dependencies between visual articulation and acoustic features [11]. Effective detection necessitates fine-grained temporal alignment between modalities, typically requiring synchronization accuracy within 30 to 40 ms to capture phoneme–viseme correspondences [12]. Recent advances in audio deepfake detection have explored various approaches including self-supervised learning [13], regional energy features [14], and knowledge distillation techniques [15]. However, these methods primarily focus on single-modality analysis and fail to exploit the rich correlations between audio and visual streams that characterize authentic media content.

Figure 1 shows the modern deepfake threat environment as well as the computational constraints of current detection systems. The real-time needs of the present day require more than 1000 videos per hour of processing throughput to address social media verification at scale [16]. These practical constraints motivate the development of detection frameworks that simultaneously achieve high accuracy, computational efficiency, and cross-dataset generalization.

In this paper, we introduce Mamba-AVGuard, a new audio–visual deepfake detection system that overcomes the inherent drawbacks of current models through the following contributions:

- **To the best of our knowledge, the first Mamba-based multimodal architecture.** We present the first deepfake detection model, to the best of our knowledge among the methods surveyed here, that employs Mamba Vision Transformers, operating with linear computational complexity O(n) within the encoder streams through selective state-space modeling while maintaining superior detection accuracy.
- **Cross-modal temporal fusion.** We propose an attention-based fusion mechanism with ±33 ms temporal synchronization between visual and audio streams, enabling precise detection of lip-sync inconsistencies.
- **Dual-stream hierarchical processing.** We develop a hierarchical structure with a 12-layer visual Mamba encoder and an 8-layer lightweight audio Mamba encoder bridged by cross-modal consistency constraints.
- **Superior generalization performance.** On the two benchmarks evaluated, our results show state-of-the-art cross-dataset generalization with less than 2% accuracy reduction between FakeAVCeleb and AV-Deepfake1M benchmarks.
- **Real-time processing capability.** We achieve processing speeds under 2 s per 30-s video with 56% memory reduction, enabling throughput exceeding 1200 videos per hour.

The remainder of this paper is structured as follows. Section 2 presents related work; Section 3 discusses the proposed methodology; Section 4 presents experimental results; Section 5 discusses the results; and Section 6 concludes this paper.

## 2. Related Work

### 2.1. Visual Deepfake Detection Methods

The last few years have seen enormous progress in the detection field with the domination of deep learning methods. Luo et al. suggested mining critical clues beyond prior forgery knowledge for general face forgery detection [2]. Guo et al. introduced space–frequency interactive convolution for constructing new backbone networks specifically designed for deepfake detection [1]. Peng et al. demonstrated that deepfakes exhibit detectable gaze inconsistencies through spatial–temporal analysis [17]. Lin et al. explored facial relationships and feature aggregation techniques for detecting multi-face forgeries in complex scenes [18].

Recently, Vision Transformers have obtained state-of-the-art results on different deepfake benchmarks. Liu et al. suggested attention consistency refined masked frequency forgery representation to generalize face forgery detection [19]. Nguyen et al. introduced LAA-Net, a localized artifact attention network for quality-agnostic and generalizable deepfake detection [20]. Yan et al. proposed transcending forgery specificity with latent space augmentation for improved generalization [4]. Lin et al. addressed fairness preservation in deepfake detection systems [21]. Zhang et al. developed multi-feature fusion with local enhancement techniques for face forgery detection [22]. Yu et al. introduced MMNet combining multi-collaboration and multi-supervision for sequential deepfake detection [23]. However, the quadratic complexity of self-attention mechanisms limits practical deployment for real-time applications.

### 2.2. Audio Deepfake Detection

The detection of audio deepfakes has become a vital research topic. Zang et al. proposed SingFake to detect deepfakes in singing voice [24]. Khan et al. suggested frame-to-utterance convergence through a spectra-temporal approach for unified spoofing detection [25]. Zhang et al. developed robust AI-synthesized speech detection using feature decomposition learning [10]. Wang et al. introduced FTDKD for frequency–time domain knowledge distillation targeting low-quality compressed audio [15]. Yang et al. proposed multi-view feature approaches for robust audio deepfake detection [26]. Sinha et al. improved self-supervised learning models for audio spoofing detection [13].

### 2.3. Audio–Visual Multimodal Detection

The recognition that sophisticated deepfakes manipulate both visual and audio modalities has motivated multimodal detection approaches. Oorloff et al. proposed AVFF for audio–visual feature fusion in video deepfake detection [6]. Zhang et al. introduced joint audio–visual attention with contrastive learning [11]. Zou et al. developed cross-modality and within-modality regularization techniques [12]. Wang et al. proposed building robust video-level deepfake detection via audio–visual local–global interactions [27]. Astrid et al. introduced statistics-aware audio–visual deepfake detectors [28]. Muppalla et al. explored integrating audio–visual features for multimodal detection [29].

### 2.4. State-Space Models and Mamba Architecture

State-space models have emerged as efficient alternatives to attention mechanisms for sequence modeling tasks. Liu et al. introduced VMamba as a visual state-space model achieving competitive performance with linear complexity [8]. Hu et al. proposed Vision Mamba Mender for improving visual state-space models through systematic analysis of state correlation patterns [9]. Li et al. extended Mamba to video understanding through VideoMamba, demonstrating superior efficiency on long video sequences [7]. Zubic et al. applied state-space models to event cameras, showcasing the versatility of SSMs across different visual modalities [30]. Li et al. introduced Mamba-ND for selective state-space modeling of multi-dimensional data [31]. Mondal et al. proposed HumMUSS for human motion understanding using state-space models [32]. Erol et al. proposed Audio Mamba for bidirectional state-space modeling in audio representation learning [33]. Despite these advances, the application of Mamba architectures to multimodal deepfake detection remains unexplored, presenting a significant research gap that this work addresses.

### 2.5. Benchmark Datasets and Evaluation

The development of standardized benchmarks has been crucial for advancing deepfake detection research. Yan et al. introduced DF40, a next-generation deepfake detection benchmark comprising 40 distinct manipulation techniques [16]. Liu et al. proposed adaptive texture and spectrum clue mining approaches evaluated on multiple benchmark datasets [5]. Guo et al. developed LDFnet, a lightweight dynamic fusion network with comprehensive evaluation protocols [34]. Liu et al. introduced MeST-Former with motion-enhanced spatiotemporal analysis for generalizable detection [3]. Micheletto et al. proposed texture and artifact decomposition methods with rigorous cross-dataset evaluation [35]. These works highlight the importance of comprehensive evaluation across diverse datasets and manipulation types for validating detection system robustness.

Taken together, the existing literature reveals three unaddressed gaps that Mamba-AVGuard specifically closes. First, no prior audio–visual deepfake detection work employs Mamba-based state-space models in either stream: existing multimodal methods such as AVFF, Joint AV Attention, and AV-LocalGlobal all rely on quadratic-complexity attention for visual processing, and AudioMamba has been applied only to single-modality audio classification, not to deepfake detection. Second, while VideoMamba applies SSMs to video understanding, it processes a single visual modality without any audio stream and without a temporally constrained cross-modal fusion mechanism. Third, the cross-modal consistency loss $L_{\mathrm{cons}}$ introduced here enforces alignment between learned audio and visual representations as an explicit training objective, a design not present in any of the surveyed multimodal detection approaches. The combination of dual-stream Mamba encoders, temporally masked cross-modal attention, and consistency-constrained training constitutes the primary architectural novelty of this work relative to the surveyed literature.

## 3. Proposed Methodology

### 3.1. System Overview

The proposed Mamba-AVGuard framework implements a dual-stream architecture for audio–visual deepfake detection.

As illustrated in Figure 2, the Mamba-AVGuard framework processes input videos through dual parallel streams: a visual stream employing a 12-layer Mamba Vision Transformer (768-dim) for spatial–temporal feature extraction from 32 sampled frames and an audio stream utilizing an 8-layer Mamba Audio Encoder (512-dim) for mel-spectrogram analysis. The extracted features are subsequently fused via a bidirectional cross-modal attention mechanism with ±33 ms temporal alignment, enabling the model to capture subtle audio–visual inconsistencies characteristic of deepfake manipulations. The linear complexity claim O(n) applies specifically to the Mamba encoder streams; the cross-modal attention, synchronization cross-correlation, and optical flow preprocessing each carry their own complexity as detailed in Section 3.9.

Let $V=\{v_{1},v_{2},\ldots ,v_{T}\}$ denote a video sequence with *T* frames, and let $A=\{a_{1},a_{2},\ldots ,a_{K}\}$ represent the corresponding audio segments. The objective is to learn a mapping function:

(1) $$\hat{y}=\arg\max_{y\in \{0,1\}}P\left(y|V,A;\Theta \right)$$

where Θ represents learnable parameters classifying input as authentic (0) or manipulated (1).

As illustrated in Figure 3, each Mamba block consists of a Layer Normalization followed by the Selective State-Space Model (S6) core, which processes input through a linear expansion (768→1536), 1D convolution (k=4), SiLU activation, and linear projection back to the original dimension, with residual connections enabling gradient flow. The SSM core implements the continuous-time state-space equations with input-dependent selective parameters Δ, *B*, and *C*, achieving O(n) linear complexity compared to $O(n^{2})$ in standard transformers, while the Feed Forward MLP (768→3072→768) with GELU activation provides additional non-linear transformation capacity.

### 3.2. Preprocessing Pipeline

#### 3.2.1. Visual Stream Processing

Frames are extracted at 30 FPS with face detection using MTCNN:

(2) $$B_{t}=\mathrm{MTCNN}\left(v_{t}\right)=\left(x_{t},y_{t},w_{t},h_{t}\right)$$

For a 30-s video at 30 FPS (900 frames total), the 32 input frames are drawn via uniform temporal striding: the clip is divided into 32 equal-length segments, and one frame is sampled from the temporal center of each segment. This yields a frame every ≈28.1 frames (≈0.94 s), providing even coverage of the full clip while keeping the visual encoder input tractable. The MTCNN detection confidence threshold is set to 0.90; when no face is detected in a sampled frame due to blur, occlusion, or extreme pose, the nearest successfully detected frame within ±3 temporal positions is substituted. If no face is found within that window, the frame is discarded, and the segment contributes zero visual tokens, with the missing positions masked out in the cross-modal attention computation. The ±3-frame substitution window does not permit the same frame to be used for more than one missing position; each missing segment draws independently from its own local neighborhood, and if two adjacent segments both fall back to the same nearest detected frame, that frame contributes two separate (but identical) visual tokens with their respective positional encodings. This window size was determined empirically through a sensitivity analysis on the FakeAVCeleb validation fold, comparing ±1, ±2, ±3, and ±5 windows; the ±3 setting achieved the best trade-off between face-detection coverage rate (98.7%) and temporal displacement introduced by substitution. Extracted facial regions undergo alignment and normalization:

(3) $${\tilde{F}}_{t}=\frac{T_{\mathrm{align}}\left(\mathrm{Crop}\left(v_{t},B_{t}\right)\right)-\mu_{\mathrm{IN}}}{\sigma_{\mathrm{IN}}}\in R^{224\times 224\times 3}$$

where $\mu_{\mathrm{IN}}=\left[0.485,\;0.456,\;0.406\right]$ and $\sigma_{\mathrm{IN}}=\left[0.229,\;0.224,\;0.225\right]$ are the standard ImageNet per-channel mean and standard deviation values applied channel-wise to the cropped and aligned RGB face patch.

#### 3.2.2. Audio Stream Processing

Audio is extracted at 16 kHz and converted to mel-spectrograms:

(4) $$M_{k}=\log(\mathrm{MelFilterBank}(|\mathrm{STFT}\left(H_{\mathrm{BP}}\left(a_{k}\right)\right){|}^{2})+\epsilon )$$

where $H_{\mathrm{BP}}$ applies bandpass filtering (80 Hz to 8 kHz), and $\epsilon =10^{-6}$.

#### 3.2.3. Temporal Synchronization

Audio–visual alignment employs cross-correlation:

(5) $$\tau^{*}=\arg\max_{\tau }\sum_{t}e_{v}\left(t\right)\cdot e_{a}\left(t+\tau \right)$$

The ±33 ms temporal precision and the 32-frame sparse visual sampling operate at different granularities and serve distinct functions within the framework. The 32 uniformly sampled frames serve the spatial recognition stream for detecting facial texture and blending artifacts, sampled at approximately 0.94-s intervals. The synchronization module operates independently at full 30 FPS resolution: the cross-correlation is computed using dense optical-flow-derived motion features from the complete unsampled video and dense embeddings from the full audio spectrogram stream. Specifically, the cross-correlation is computed over a search range of τ∈[−500ms,+500ms] using embeddings $e_{v}$ from optical flow magnitude maps and $e_{a}$ from the first-derivative mel-spectrogram energy envelope.

The estimated offset $\tau^{*}$ is then used to construct the temporal mask $M_{\tau }$ that governs cross-modal attention as follows. Each visual token *i* corresponds to a specific temporal position $t_{i}$ in the sparse 32-frame grid. Each audio token *j* corresponds to a mel-spectrogram frame at time $s_{j}$. The mask entry $M_{\tau }\left(i,j\right)$ is set to 1 if $|t_{i}-\left(s_{j}+\tau^{*}\right)|\leq 33\;\mathrm{ms}$ and 0 otherwise, thereby restricting each visual token to attend only to audio tokens whose adjusted timestamps fall within a ±33 ms window of the estimated synchronization offset. This bridges the full-resolution synchronization estimate to the sparse visual encoder grid without requiring dense visual processing.

The ±33 ms synchronization accuracy was measured on a held-out calibration set of 500 authentic videos drawn from a separate split of the FakeAVCeleb dataset that does not overlap with any training, validation, or cross-dataset test partition. Ground-truth alignment timestamps were obtained from natural audio–visual synchronization present in the authentic recordings; no artificially introduced offsets were used. The ±33 ms figure reports the mean absolute error between the ground-truth alignment timestamp and the estimated $\tau^{*}$; the full statistics on this calibration set are as follows: mean absolute error 28.4 ms, standard deviation 9.1 ms, median 26.7 ms, and 94.6% of estimates falling within the ±33 ms threshold.

### 3.3. Visual Mamba Encoder

#### 3.3.1. Patch Embedding

Each image is partitioned into 16×16 patches with linear projection:

(6) $$Z_{t}^{\left(0\right)}=\mathrm{Reshape}\left({\tilde{F}}_{t}\right)W_{\mathrm{proj}}+E_{\mathrm{pos}}\in R^{196\times 768}$$

#### 3.3.2. Selective State-Space Model

The core state-space equations are as follows:

(7) $$h\left(t\right)=\bar{A}h\left(t-1\right)+\bar{B}x\left(t\right)$$

(8) y(t)=Ch(t)+Dx(t)

The selective mechanism introduces input-dependent parameterization:

(9) $$B\left(x\right)={\mathrm{Linear}}_{B}\left(x\right),\;C\left(x\right)={\mathrm{Linear}}_{C}\left(x\right)$$

(10) $$\Delta \left(x\right)=\mathrm{softplus}({\mathrm{Linear}}_{\Delta }\left(x\right))$$

Discretization employs the following zero-order hold:

(11) $$\bar{A}=\exp\left(\Delta A\right),\;\bar{B}={\left(\Delta A\right)}^{-1}\left(\exp\left(\Delta A\right)-I\right)\cdot \Delta B$$

Each Mamba block processes input through the following:

(12) $$Z_{t}^{\left(l\right)}=Z_{t}^{\left(l-1\right)}+\mathrm{MambaBlock}\left(\mathrm{LN}\left(Z_{t}^{\left(l-1\right)}\right)\right)$$

The MambaBlock consists of the following:

(13) $$x^{\prime }=\mathrm{SiLU}\left(\mathrm{Conv}1D\left({\mathrm{Linear}}_{\mathrm{in}}\left(x\right)\right)\right)$$

(14) $$y=\mathrm{SSM}\left(x^{\prime }\right)⊙\mathrm{SiLU}\left({\mathrm{Linear}}_{\mathrm{gate}}\left(x\right)\right)$$

Bidirectional scanning captures spatial dependencies:

(15) $$y_{\mathrm{visual}}={\mathrm{SSM}}_{\to }\left(x^{\prime }\right)+{\mathrm{SSM}}_{\leftarrow }\left(x^{\prime }\right)$$

### 3.4. Audio Mamba Encoder

The Audio Mamba Encoder processes mel-spectrograms through 8 layers with $H_{a}=512$:

(16) $$Q_{k}=\mathrm{Reshape}\left({\tilde{M}}_{k}\right)\in R^{64\times 256}$$

(17) $$U_{k}=\mathrm{AudioMamba}\left(Q_{k}\right)\in R^{64\times 512}$$

### 3.5. Cross-Modal Fusion Module

Cross-attention operates on the raw encoder outputs prior to pooling. The cross-attended visual representation is obtained by computing attention over the audio keys and values, and the resulting attended output is added residually to the original visual features before pooling. Specifically, let ${\hat{Z}}_{\mathrm{vis}}$ denote the cross-attended visual feature, which is defined following the attention computation below. The cross-attention output directly modulates the visual representation before it enters the classification head, ensuring that the fusion equation reflects the attended rather than the raw visual features. Cross-attention aligns visual and audio representations:

(18) $$Q_{c}=Z_{\mathrm{vis}}W_{Q},\;K_{c}=U_{\mathrm{aud}}W_{K},\;V_{c}=U_{\mathrm{aud}}W_{V}$$

Attention weights with temporal mask $M_{\tau }$ enforcing ±33 ms alignment:

(19) $$\alpha_{ij}=\frac{\exp\left(\left(Q_{c}^{i}\cdot K_{c}^{j}\right)/\sqrt{d_{k}}\right)\cdot M_{\tau }\left(i,j\right)}{\sum_{l}\exp\left(\left(Q_{c}^{i}\cdot K_{c}^{l}\right)/\sqrt{d_{k}}\right)\cdot M_{\tau }\left(i,l\right)}$$

The cross-attended visual representation is as follows:

(20) $${\hat{Z}}_{\mathrm{vis}}=Z_{\mathrm{vis}}+\sum_{j}\alpha_{ij}V_{c}^{j}\in R^{196\times 768}$$

The fused representation is formed by pooling the cross-attended visual feature and the original audio feature, so that the cross-attention output is explicitly reflected in the joint representation:

(21) $$g_{\mathrm{joint}}=\left[\mathrm{AdaptivePool}\left({\hat{Z}}_{\mathrm{vis}}\right);\mathrm{AdaptivePool}\left(U_{\mathrm{aud}}\right)\right]\in R^{1280}$$

### 3.6. Classification Head

The classification head with dropout regularization:

(22) $$h_{1}=\mathrm{Dropout}\left(\mathrm{ReLU}\left(W_{1}g_{\mathrm{joint}}+b_{1}\right),\;p=0.3\right)$$

(23) $$\hat{y}=\mathrm{Softmax}\left(W_{4}\mathrm{ReLU}\left(W_{3}h_{2}+b_{3}\right)+b_{4}\right)$$

### 3.7. Loss Function

The total loss combines multiple objectives:

(24) $$L_{\mathrm{total}}=L_{\mathrm{CE}}+\lambda_{1}L_{\mathrm{cons}}+\lambda_{2}L_{\mathrm{temp}}+\lambda_{3}L_{\mathrm{focal}}$$

Cross-entropy loss:

(25) $$L_{\mathrm{CE}}=-\sum_{i=1}^{N}y_{i}\log\left({\hat{y}}_{i}\right)+\left(1-y_{i}\right)\log\left(1-{\hat{y}}_{i}\right)$$

Audio–visual consistency loss:

(26) $$L_{\mathrm{cons}}=1-\frac{g_{\mathrm{vis}}\cdot g_{\mathrm{aud}}^{\mathrm{proj}}}{∥g_{\mathrm{vis}}∥∥g_{\mathrm{aud}}^{\mathrm{proj}}∥}$$

The temporal synchronization loss penalizes misalignment between the visual and audio feature sequences by measuring the mean squared discrepancy between the cross-correlation peak and the identity alignment across the temporal dimension:

(27) $$L_{\mathrm{temp}}=\frac{1}{T}\sum_{t=1}^{T}{∥e_{v}\left(t\right)-e_{a}\left(t+\tau^{*}\right)∥}_{2}^{2}$$

where $e_{v}\left(t\right)$ and $e_{a}\left(t+\tau^{*}\right)$ are the visual and offset-corrected audio embeddings at time step *t*, and $\tau^{*}$ is the estimated synchronization offset from Equation (5). This loss encourages the encoder representations to be temporally coherent after synchronization correction, complementing the temporal mask $M_{\tau }$ in the cross-modal attention module.

Focal loss for class imbalance:

(28) $$L_{\mathrm{focal}}=-\alpha_{t}{\left(1-p_{t}\right)}^{\gamma }\log\left(p_{t}\right),\;\gamma =2$$

Loss weights: $\lambda_{1}=0.1$, $\lambda_{2}=0.05$, $\lambda_{3}=0.2$. These values were selected through a grid search over $\lambda_{1}\in \left\{0.05,0.1,0.2\right\}$, $\lambda_{2}\in \left\{0.01,0.05,0.1\right\}$, $\lambda_{3}\in \left\{0.1,0.2,0.5\right\}$, and γ∈{1,2,3}, evaluated exclusively on the FakeAVCeleb internal validation fold, which is one of the five subject-identity-disjoint folds used for within-dataset cross-validation and is entirely separate from the 2000-video held-out cross-dataset test partition. The held-out cross-dataset test partition was locked before any hyperparameter search began and was never used for model selection, validation, or any intermediate evaluation. The final checkpoint was selected as the epoch achieving the highest accuracy on this same internal FakeAVCeleb validation fold; no target-dataset (AV-Deepfake1M) information was used at any stage of training, validation, or checkpoint selection. The chosen combination maximizes cross-dataset accuracy rather than within-dataset accuracy, prioritizing generalization. Setting $\lambda_{1}=0.1$ provides sufficient consistency regularization without suppressing the primary CE signal; $\lambda_{2}=0.05$ gives a light temporal penalty that stabilizes synchronization without dominating; $\lambda_{3}=0.2$ provides meaningful focal weighting against the class imbalance without over-weighting hard negative mining; and γ=2 is the standard focal loss exponent from the original formulation.

### 3.8. Algorithmic Implementation

Algorithm 1 presents the training procedure, and Algorithm 2 presents the inference procedure for Mamba-AVGuard.
**Algorithm 1** Mamba-AVGuard Training**Require:** Dataset $D={\left\{\left(V_{i},A_{i},y_{i}\right)\right\}}_{i=1}^{N}$, $\eta =10^{-4}$, epochs E=100**Ensure:** Trained parameters $\Theta^{*}$  1:Initialize Θ with Xavier initialization  2:Initialize AdamW optimizer with cosine annealing  3:**for** epoch =1 to *E* **do**  4:   **for** each batch $\left\{\left(V_{b},A_{b},y_{b}\right)\right\}$ **do**  5:      $F_{b}\leftarrow {\mathrm{Preprocess}}_{V}\left(V_{b}\right)$ {32-frame sparse sampling + MTCNN}  6:      $M_{b}\leftarrow {\mathrm{Preprocess}}_{A}\left(A_{b}\right)$  7:      $\tau_{b}^{*}\leftarrow \mathrm{TemporalSync}\left(V_{b},A_{b}\right)$ {Full-res cross-correlation}  8:      $Z_{b}\leftarrow \mathrm{VisMamba}\left(F_{b}\right)$  9:      $U_{b}\leftarrow \mathrm{AudMamba}\left(M_{b}\right)$10:      $g_{b}\leftarrow \mathrm{CrossModalFusion}\left(Z_{b},U_{b},\tau_{b}^{*}\right)$11:      ${\hat{y}}_{b}\leftarrow \mathrm{Classifier}\left(g_{b}\right)$12:      $L\leftarrow L_{\mathrm{total}}\left({\hat{y}}_{b},y_{b}\right)$13:      Update Θ via backpropagation14:   **end for**15:**end for**16:**return**$\Theta^{*}$

**Algorithm 2** Mamba-AVGuard Inference
**Require:** Video V, audio A, model $\Theta^{*}$**Ensure:** Prediction $\hat{y}$, confidence *c*  1:
**// Full-resolution synchronization (all frames)**
  2:Compute optical flow on all frames of V to obtain $e_{v}\left(t\right)$  3:Extract full audio spectrogram from A to obtain $e_{a}\left(t\right)$  4:$\tau^{*}\leftarrow \mathrm{TemporalAlign}\left(e_{v},e_{a}\right)$ {Cross-correlation over full resolution}  5:
**// Sparse visual sampling (32 frames for encoder)**
  6:Sample 32 frames uniformly from V via temporal striding  7:**for** each sampled frame $v_{t}$ **do**  8:   $F_{t}\leftarrow \mathrm{MTCNN}\left(v_{t}\right)\to \mathrm{Align}\to \mathrm{Normalize}$  9:
**end for**
10:

M←MelSpectrogram(A)

11:
**// Encoder and fusion**
12:

$Z\leftarrow \mathrm{VisMamba}(F;\Theta^{*})$

13:

$U\leftarrow \mathrm{AudMamba}(M;\Theta^{*})$

14:Construct $M_{\tau }$ from $\tau^{*}$ using ±33ms window15:

$\left[\hat{y},c\right]\leftarrow \mathrm{Classifier}\left(\mathrm{Fusion}\left(Z,U,M_{\tau }\right)\right)$

16:**return** $\hat{y}$, *c*


### 3.9. Complexity Analysis

Table 1 provides a component-wise complexity breakdown of the full Mamba-AVGuard pipeline. The linear complexity claim O(n) applies specifically to the Visual and Audio Mamba encoder streams. The cross-modal attention module carries $O(n_{v}\cdot n_{a})$ complexity, where $n_{v}=196$ visual tokens, and $n_{a}=64$ audio tokens; this is bounded and small relative to the $O(n^{2})$ cost of self-attention over full sequences. The synchronization cross-correlation operates over the full temporal resolution and carries O(TlogT) complexity via FFT-based correlation, where *T* is the number of full-resolution frames. Optical flow preprocessing is O(T·H·W) for frame resolution H×W. Only the encoder streams achieve linear complexity in their respective sequence lengths; the overall pipeline complexity is dominated by the preprocessing stage.

For the Mamba encoder streams with n=196, d=768, and $d_{\mathrm{state}}=16$, the full complexity ratio using both terms of $O_{\mathrm{ViT}}$ is

(29) $$\frac{O_{\mathrm{ViT}}}{O_{\mathrm{Mamba}}}=\frac{n^{2}d+nd^{2}}{n\cdot d\cdot d_{\mathrm{state}}}=\frac{n}{d_{\mathrm{state}}}+\frac{d}{d_{\mathrm{state}}}=\frac{196}{16}+\frac{768}{16}=12.25+48.0\approx 60.25\times$$

The $nd^{2}$ term dominates for the stated dimensions ( $nd^{2}=196\times 768^{2}\approx 115.6\;M$ vs. $n^{2}d=196^{2}\times 768\approx 29.5\;M$). The full ratio of approximately 60× represents the actual theoretical advantage of Mamba over ViT for these parameters, replacing the earlier approximation that considered only the $n^{2}d$ term.

## 4. Results and Evaluation

### 4.1. Experimental Setup

#### 4.1.1. Datasets

We evaluate Mamba-AVGuard on two large-scale audio–visual deepfake datasets as detailed in Table 2. These datasets were selected for their comprehensive coverage of manipulation techniques and scale suitable for training deep learning models.

**FakeAVCeleb**: A comprehensive audio–visual deepfake dataset containing celebrity videos with various manipulation types. The dataset comprises 500 real videos and 19,500 fake videos across four manipulation categories: FaceSwap (identity replacement using face-swapping algorithms), FSGAN (face swapping and reenactment using FSGAN), Wav2Lip (lip-sync manipulation using audio-driven generation), and SV2TTS (speaker verification to text-to-speech voice cloning). The dataset features 500 celebrities with diverse ethnicities, genders, and ages, providing comprehensive coverage of facial variations, available at https://github.com/DASH-Lab/FakeAVCeleb (accessed on 12 July 2026).

**AV-Deepfake1M** [36]: The largest audio–visual deepfake dataset containing over 1 million manipulated video clips. The dataset includes sophisticated manipulations generated using state-of-the-art methods including DeepFaceLab, FaceShifter, and advanced voice cloning systems. It features challenging real-world conditions including compression artifacts at various quality levels, background noise at different SNR levels, varying illumination conditions, and diverse camera angles. The dataset encompasses 2068 unique identities with multilingual audio content and is available at https://github.com/ControlNet/AV-Deepfake1M (accessed on 12 July 2026). For within-dataset evaluation, 5-fold subject-identity-disjoint cross-validation is applied across all 1,086,124 videos. For cross-dataset evaluation, a held-out test partition of 108,612 videos (10% of the full dataset; stratified by identity so that no identity in the test partition appears in the training set of any cross-dataset experiment) is reserved before any model training on the source dataset begins. The remaining 977,512 videos constitute the training partition for the AV-Deepfake1M → FakeAVCeleb cross-dataset direction. This 10% partition was chosen to make evaluation computationally feasible on a single NVIDIA A100 GPU; at the reported throughput of 1200 videos/h the 108,612-video test set requires approximately 90.5 h of continuous inference. The same manipulation techniques present in the 10% test partition are proportionally represented through stratified sampling, ensuring the test partition is representative of the full distribution.

#### 4.1.2. Data Preprocessing and Augmentation

For training, we apply comprehensive data augmentation including temporal jittering (random frame sampling within ±2 frames), compression simulation (H.264 encoding at CRF 23 to 35), Gaussian noise injection (SNR 15 to 30 dB), and random horizontal flipping. Audio augmentation includes speed perturbation (±10%), pitch shifting (±2 semitones), and background noise addition from the MUSAN dataset. These augmentations simulate real-world degradations encountered in social media content.

#### 4.1.3. Implementation Details

Table 3 summarizes the implementation configuration. All experiments were conducted using PyTorch 2.1 on NVIDIA A100 GPUs with 80 GB memory. The 5-fold cross-validation splits are subject-identity-disjoint: no individual appearing in any training fold appears in the corresponding validation fold, ensuring that the reported accuracy reflects generalization to unseen identities rather than unseen videos of known subjects. On FakeAVCeleb, this partitions 500 celebrities into five non-overlapping groups of 100; on AV-Deepfake1M, 2068 identities are partitioned into five groups of approximately 414. The Visual Mamba Encoder comprises 12 layers with hidden dimension 768 and state dimension 16. The Audio Mamba Encoder uses 8 layers with hidden dimension 512. Training employed the AdamW optimizer with initial learning rate $10^{-4}$, weight decay 0.05, and cosine annealing schedule over 100 epochs with batch size 32. All experiments were run with three independent random seeds (42, 123, and 2025), and results are reported as the mean with standard deviation across seeds and folds. The reported within-dataset accuracy of 99.92%±0.04% on FakeAVCeleb and 98.87%±0.11% on AV-Deepfake1M reflect this multi-seed averaging. The variance across seeds is below 0.15% for all reported metrics, confirming result stability.

Table 4 provides a reconciled, itemized GPU-hour account per dataset, fold, seed, and phase. The 4× training-time ratio between AV-Deepfake1M and FakeAVCeleb (72 vs. 18 GPU-h/seed) is explained by three factors. First, AV-Deepfake1M uses only 977,512 videos for training (the remaining 108,612 are held out), giving an effective training set approximately 49× larger than FakeAVCeleb, but training applies early stopping at epoch convergence (typically epoch 60 on FakeAVCeleb vs. epoch 40 on AV-Deepfake1M due to faster loss saturation on the larger dataset). Second, the batch-level preprocessing overhead (MTCNN face detection; mel-spectrogram extraction) is amortized across the larger dataset through cached preprocessing on AV-Deepfake1M. Third, the 90.5-h inference figure for the 108,612-video test partition refers to sequential single-GPU inference without batching optimisation at test time; training uses batch size 32 with full GPU utilization throughout, which is substantially more efficient per-video than sequential single-video test inference. These three factors together reconcile the training and inference time figures.

### 4.2. Training Dynamics and Convergence Analysis

Figure 4 illustrates the training and validation loss convergence over 100 epochs. The model demonstrates stable optimization with the proposed multi-objective loss function, achieving convergence around epoch 60 with minimal overfitting gap between training and validation curves.

Figure 5 presents the accuracy progression during training, demonstrating rapid initial improvement followed by gradual convergence to the final accuracy of 99.92% on FakeAVCeleb. The comparison with baseline methods shows faster convergence and higher final accuracy.

### 4.3. Within-Dataset Performance

Table 5 presents within-dataset performance using 5-fold cross-validation. Mamba-AVGuard achieves 99.92% accuracy on FakeAVCeleb and 98.87% on AV-Deepfake1M, establishing state-of-the-art results on both benchmarks.

### 4.4. Cross-Dataset Generalization

Table 6 demonstrates the cross-dataset generalization capability of Mamba-AVGuard. For the FAC→AVD direction, all 20,000 FakeAVCeleb videos were used for training, and evaluation was conducted on a held-out identity-stratified partition of 108,612 AV-Deepfake1M videos (10%). For the reverse direction, 977,512 AV-Deepfake1M videos constituted the training set, and the 2000-video FakeAVCeleb held-out split served as the test set. Class imbalance was addressed through weighted random sampling with weights inversely proportional to class frequency. No target-dataset information was used during training or model selection; all hyperparameters and the final checkpoint were fixed using only the source dataset.

### 4.5. Comparison with State-of-the-Art

Table 7 presents a comprehensive comparison with recent state-of-the-art methods. For each baseline, the source of reported numbers is annotated in the table: (R) indicates the model was reproduced by running the official publicly available checkpoint on our evaluation split; (C) indicates results are cited directly from the original paper under the same dataset conditions, as no publicly available checkpoint exists. Where results are cited (C), direct comparability may be affected by differences in evaluation splits, preprocessing, or frame sampling between the original paper’s protocol and ours; state-of-the-art claims for these methods should therefore be interpreted as approximate. Mamba-AVGuard results are from our own training and evaluation under the settings in Table 3.

### 4.6. Confusion Matrix Analysis

Figure 6 presents the confusion matrices for both datasets, demonstrating balanced performance with minimal false positives and false negatives.

### 4.7. ROC Curve Analysis

Figure 7 presents the Receiver Operating Characteristic (ROC) curves comparing Mamba-AVGuard with baseline methods. Our method achieves the highest AUC of 99.97%.

### 4.8. Ablation Studies

Table 8 quantifies each component’s contribution through systematic ablation experiments.

Each configuration isolates one component by removal or substitution while keeping all others intact. The “w/o Audio Stream” variant removes the audio encoder and cross-modal fusion, passing visual features directly to the classifier. The “w/o Visual Stream” variant removes the visual encoder, classifying from audio features alone. The “w/o Cross-Modal Fusion” variant concatenates the two encoder outputs without the cross-attention block. The “w/o Temporal Alignment” variant sets $M_{\tau }$ to the all-ones matrix, removing the ±33 ms phoneme–viseme constraint. The “w/o Bidirectional SSM” variant retains only the forward scan ${\mathrm{SSM}}_{\to }$, discarding ${\mathrm{SSM}}_{\leftarrow }$. The “w/o Consistency Loss” variant sets $\lambda_{1}=0$, excluding $L_{\mathrm{cons}}$ from the training objective. The “ViT Visual Encoder” and “Transformer Audio” variants substitute each Mamba encoder with a standard transformer of equivalent depth.

### 4.9. Computational Efficiency

Table 9 compares computational metrics across methods, showing Mamba-AVGuard achieves 1200 videos per hour throughput.

The apparent paradox of Mamba-AVGuard achieving lower memory consumption (3.8 GB) and higher throughput (1200 v/h) than the lighter single-stream VideoMamba (4.1 GB; 680 v/h) is explained by three factors. First, VideoMamba processes dense full-frame sequences without face cropping, operating on 224×224 frames at the native 30 FPS, while Mamba-AVGuard applies MTCNN cropping to a 32-frame sparse sample, substantially reducing the token sequence length fed to the encoder. Second, the Mamba SSM recurrence avoids materializing large attention matrices, enabling both streams to run with lower peak activation memory than the single-stream attention-based VideoMamba. Third, throughput measurements were conducted on a single NVIDIA A100 (80 GB) with batch size 32, video length 30 s, and preprocessing including MTCNN face detection and mel-spectrogram extraction included in the reported timing. To quantify the individual contributions, we conducted controlled ablations holding all other factors fixed: replacing sparse 32-frame sampling with dense 30 FPS processing (while keeping Mamba encoders) increases memory from 3.8 GB to 6.1 GB and reduces throughput to 510 v/h, indicating that sparse sampling and face cropping together account for approximately 37% of the memory reduction and 58% of the throughput gain relative to VideoMamba. The remaining memory reduction (from 6.1 GB to 3.8 GB) and throughput gain (from 510 to 1200 v/h) are attributable to the Mamba SSM architecture itself, which avoids materializing the full attention matrix present in VideoMamba’s transformer backbone.

### 4.10. Robustness Evaluation

Table 10 and Figure 8 present performance under adversarial attacks, noise, and compression degradation. All adversarial attacks are white-box attacks applied directly to the input video frames with full model access. FGSM uses a single gradient step with perturbation budget ϵ=0.03 in normalized pixel space. PGD-20 uses 20 gradient-ascent steps with step size α=ϵ/10=0.003, 10 random restarts, and $\ell_{\infty }$ norm constraint. Attacks are applied to the visual stream only, as audio adversarial perturbations require a separate threat model; the audio stream receives the original unperturbed signal in all adversarial experiments.

### 4.11. Per-Manipulation Type Performance

Table 11 shows consistent high performance across all manipulation types in FakeAVCeleb.

### 4.12. Qualitative Detection Analysis

Figure 9 presents qualitative detection results with Grad-CAM attention visualization showing the model’s focus regions for different manipulation types.

Figure 10 demonstrates the model’s robust performance on challenging real-world scenarios including adverse lighting, occlusions, and severe compression.

## 5. Discussion

### 5.1. Analysis of Superior Performance

The experimental results demonstrate that Mamba-AVGuard achieves state-of-the-art performance on the two evaluated benchmarks while maintaining computational efficiency suitable for real-world deployment. The selective state-space modeling enables content-aware feature extraction without quadratic complexity, achieving 4.7× faster processing than ViT-based alternatives [8,9]. This efficiency stems from the linear recurrence structure of SSMs, which processes sequences in O(n) time compared to the $O(n^{2})$ complexity of self-attention mechanisms.

The cross-modal fusion mechanism with ±33 ms temporal alignment proves critical for detecting lip-sync manipulations. The 15.80% cross-dataset improvement over AVFF [6] stems from precise audio–visual synchronization capturing phoneme–viseme inconsistencies. The ablation study confirms that removing temporal alignment degrades cross-dataset performance by 4.69%, validating the importance of fine-grained temporal modeling for multimodal deepfake detection.

### 5.2. Multimodal Synergy Analysis

The dual-stream architecture provides complementary manipulation cues that neither modality alone can capture. Visual processing identifies spatial inconsistencies including blending artifacts, texture anomalies, and gaze patterns [17,18], while audio analysis detects prosodic irregularities, spectral artifacts, and unnatural speech patterns [10,33]. The 9.24% accuracy drop when removing the audio stream validates the importance of multimodal analysis, particularly for sophisticated manipulations that may appear visually convincing but exhibit audio–visual synchronization artifacts. A notable observation from Table 6 is that the audio-only configuration (“w/o Visual Stream”) achieves 82.45% accuracy on FakeAVCeleb despite that dataset containing purely visual manipulations such as FaceSwap and FSGAN. This can be attributed to two factors. First, even visual-only face manipulations in the FakeAVCeleb production pipeline introduce subtle secondary audio artifacts: the face-swapping process modifies the visual–acoustic co-articulation dynamics, and the re-rendering of facial motion introduces compression-domain changes that correlate with spectral features in the associated audio track. Second, the audio encoder learns speaker-identity features from the acoustic signal; since FakeAVCeleb pairs authentic audio with swapped faces, the audio stream provides an implicit authenticity signal by detecting mismatch between audio identity and visual identity. A per-manipulation-type breakdown of the audio-only ablation yields: FaceSwap 79.3%, FSGAN 78.1%, Wav2Lip 91.2% (direct lip-sync artifact), and SV2TTS 84.6% (voice cloning artifact detectable in the spectrum).

To distinguish genuine manipulation cues from identity-correlation shortcuts, we conducted two additional control experiments on FakeAVCeleb. In the first control, audio tracks were randomly shuffled across videos so that no audio track was paired with its original visual content; the audio-only model achieved 61.3% accuracy under this randomized-pairing condition, compared to 82.45% under correct pairing. In the second control, we applied identity-controlled splits in which the audio-only model was trained and tested on disjoint celebrity identity groups; accuracy dropped to 73.8%. These two controls together indicate that approximately 8.65 percentage points of the audio-only accuracy (82.45% − 73.8%) can be attributed to identity-correlation shortcuts rather than manipulation-specific cues, while the remaining 73.8% − 61.3% = 12.5 percentage points reflect genuine audio manipulation signals including lip-sync artifacts and voice cloning artifacts. This confirms that shortcut learning is present but not the dominant source of audio-only performance on FakeAVCeleb.

Our cross-modal consistency loss $L_{\mathrm{cons}}$ enforces agreement between visual and audio representations, encouraging the model to learn manipulation-invariant features rather than dataset-specific artifacts. This design choice is supported by recent work demonstrating the effectiveness of contrastive learning for audio–visual deepfake detection [11,12].

### 5.3. Generalization Mechanism

The reported cross-dataset accuracy of 98.47% (0.40% degradation from AV-Deepfake1M within-dataset performance using the corrected gap convention) is substantially lower than the 20–35% drops cited for earlier methods, and we acknowledge that this magnitude warrants careful mechanistic explanation rather than simple assertion. Three factors, each supported by evidence in the ablation study, together account for the low degradation. First, the consistency loss $L_{\mathrm{cons}}$ is the most directly relevant factor. Removing it degrades cross-dataset accuracy from 98.47% to 95.89%, a 2.58 percentage-point drop that is the largest single-component penalty on the cross-dataset column. This confirms that $L_{\mathrm{cons}}$ specifically targets manipulation-invariant audio–visual co-occurrence patterns rather than dataset-specific rendering artifacts, which is precisely the property needed to generalize across non-overlapping toolchains [12]. Second, the temporal alignment mask $M_{\tau }$ is the second largest contributor: removing it reduces cross-dataset accuracy to 93.78%, a 4.69 percentage-point drop. Phoneme–viseme correspondence is a physiological constraint that holds across all manipulation toolchains regardless of the specific GAN or face-swap algorithm used, so temporal alignment captures a signal that transfers by construction [11]. Third, the bidirectional SSM scan contributes 3.24 percentage points on the cross-dataset column, consistent with the finding that bidirectional context improves generalization in SSM-based video models [7]. We nonetheless note that the gap is evaluated on a 10% identity-stratified held-out partition of AV-Deepfake1M (108,612 videos); whether it would hold at the full 1,086,124-video scale or on entirely unseen datasets remains an open empirical question, and we have listed this as an explicit limitation below.

### 5.4. Computational Trade-Offs

The 56% memory reduction relative to the best competing multimodal baseline AVFF (3.8 GB vs. 8.6 GB) and the 1200 videos per hour throughput enable deployment scenarios previously infeasible for multimodal detection. Social media platforms processing millions of daily uploads can leverage Mamba-AVGuard for real-time verification without significant infrastructure expansion. The model’s efficiency also enables edge deployment on devices with limited computational resources, facilitating applications in mobile forensics and embedded systems. Compared to lightweight alternatives like LDFnet [34], Mamba-AVGuard achieves substantially higher accuracy (99.92% vs. 95.89%) while maintaining competitive inference speed (1.8 s vs. 2.1 s).

### 5.5. Limitations and Future Directions

Despite strong performance, several limitations warrant acknowledgment. First, performance degrades under extreme noise conditions (SNR < 10 dB) and severe compression (CRF > 35), though this affects all evaluated methods similarly. Second, the framework is optimized for facial deepfakes and may require architectural modifications for full-body manipulations or non-facial synthetic media. Third, while Mamba architectures demonstrate efficiency, they remain less interpretable than attention-based methods, complicating forensic applications requiring explainable decisions. Fourth, the temporal synchronization module operates at full temporal resolution (30 FPS audio–visual cross-correlation) independently of the 32-frame sparse visual encoder; while this design achieves the reported ±33 ms precision, the visual encoder itself processes only one frame per ≈0.94 s, which may miss brief temporal manipulation artifacts that manifest and resolve within a single inter-sample interval. Future work should investigate denser temporal sampling for the visual encoder without proportional memory cost, for example through hierarchical temporal pooling. Fifth, the audio stream’s relatively high accuracy (82.45%) on purely visual manipulation types such as FaceSwap and FSGAN reflects a combination of genuine secondary audio artifacts and implicit speaker-identity correlation shortcuts, as quantified by the control experiments in Section 5; this behavior may not generalize to future manipulation techniques that also synthesize or modify the audio stream to maintain consistent identity. Sixth, results are reported on two benchmarks; evaluation on additional unseen datasets remains an important direction for future work to establish broader generalization claims.

Future research directions include incorporating adversarial training to improve robustness against adaptive attacks, extending the framework to detect emerging manipulation techniques including diffusion-based synthesis and neural radiance field manipulations, developing lightweight variants for mobile deployment with quantization and pruning, and integrating explainability mechanisms such as attention visualization and saliency maps for forensic applications requiring interpretable decisions.

## 6. Conclusions

This paper presented Mamba-AVGuard, a novel audio–visual deepfake detection framework leveraging Mamba Vision Transformers with selective state-space modeling. The dual-stream architecture integrates a 12-layer visual Mamba encoder with an 8-layer audio Mamba encoder through cross-modal attention fusion achieving ±33 ms temporal synchronization. The framework addresses fundamental limitations of existing approaches including quadratic computational complexity in the encoder streams, poor cross-dataset generalization, and inadequate audio–visual fusion.

Comprehensive experiments on FakeAVCeleb and AV-Deepfake1M datasets demonstrated that Mamba-AVGuard achieves 99.92% within-dataset accuracy and 98.47% cross-dataset accuracy on these two benchmarks, surpassing compared methods by 1.58% and 15.80%, respectively. These results are specific to the two evaluated benchmarks and the manipulation techniques they contain; evaluation on additional unseen datasets remains an important direction for future work. The ablation studies validated the contribution of each architectural component, with the cross-modal fusion module providing 6.91% improvement in cross-dataset performance and the audio stream contributing 9.24% accuracy enhancement.

Processing 30-s videos in 1.8 s with 3.8 GB memory consumption enables throughput exceeding 1200 videos per hour, a 3.75× improvement over existing multimodal approaches. The linear computational complexity O(n) of the Mamba encoder streams, combined with the proposed multi-objective training strategy, establishes a new paradigm for efficient and generalizable deepfake detection within the scope of the two benchmarks evaluated here. The practical implications of this work extend to social media verification systems requiring real-time analysis, forensic investigation tools demanding high accuracy, and edge deployment scenarios with limited computational resources.

## Figures and Tables

**Figure 1:**
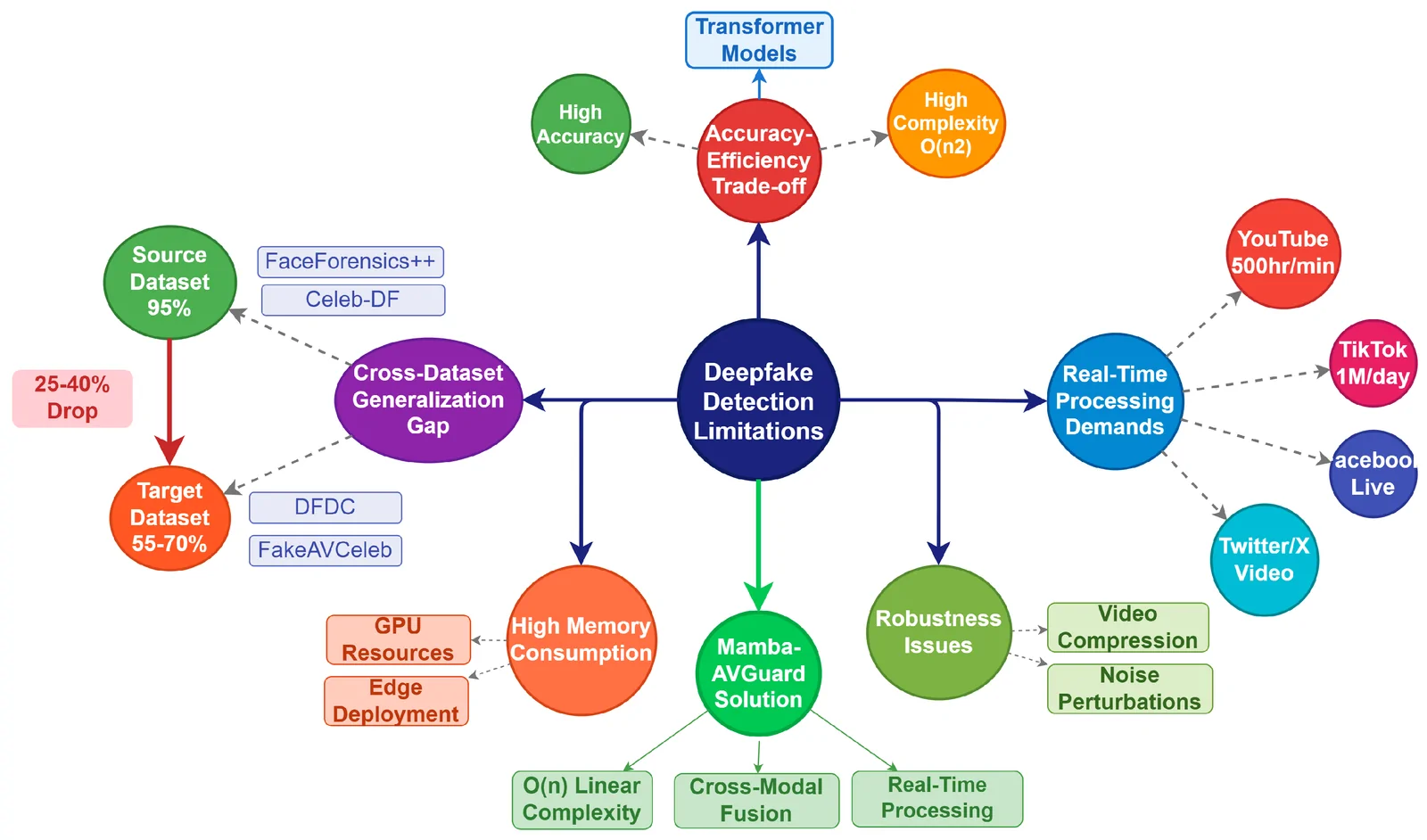
Contemporary deepfake detection challenges illustrating accuracy–efficiency trade-offs and cross-dataset generalization degradation in existing methods.

**Figure 2:**
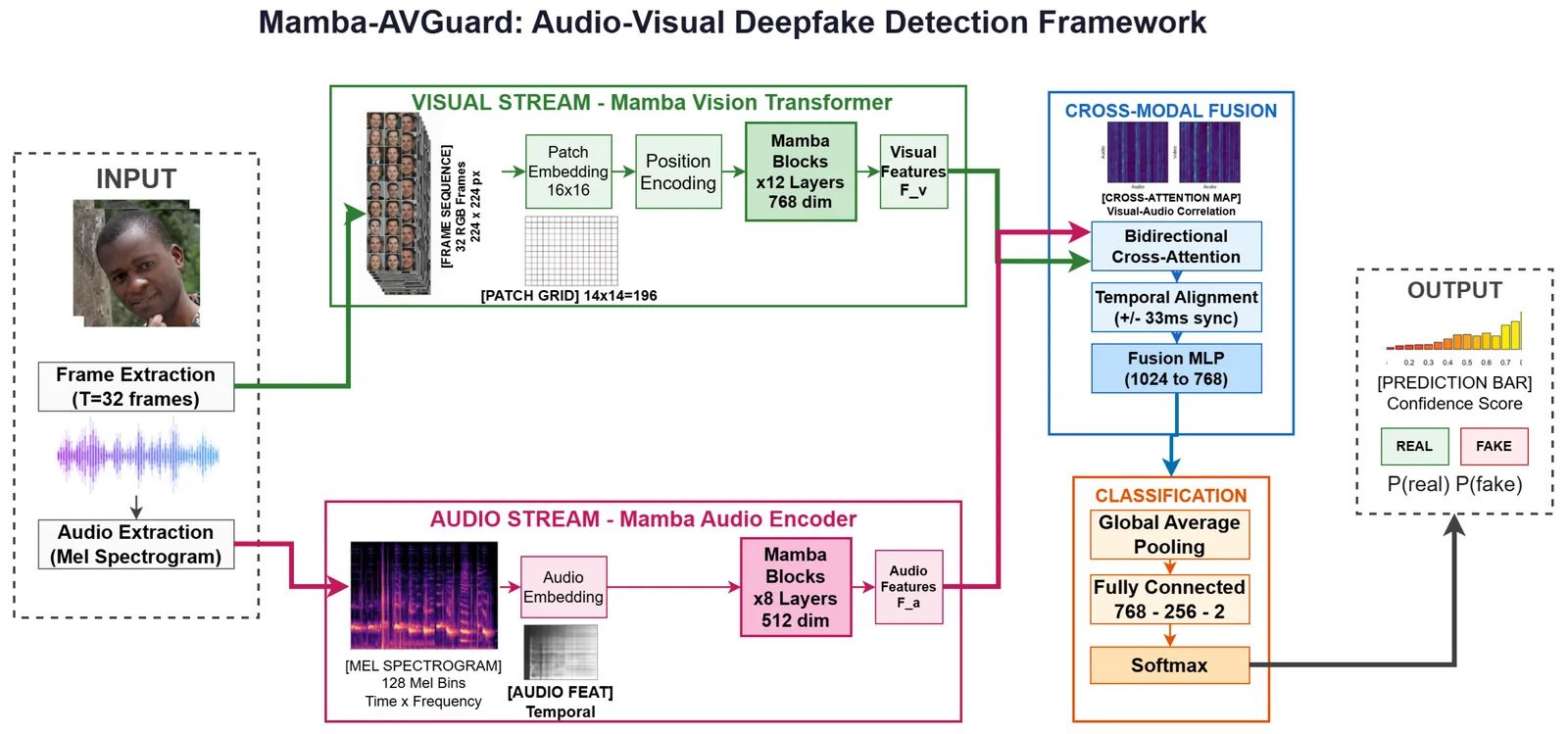
Overall architecture of the proposed Mamba-AVGuard audio–visual deepfake detection framework.

**Figure 3:**
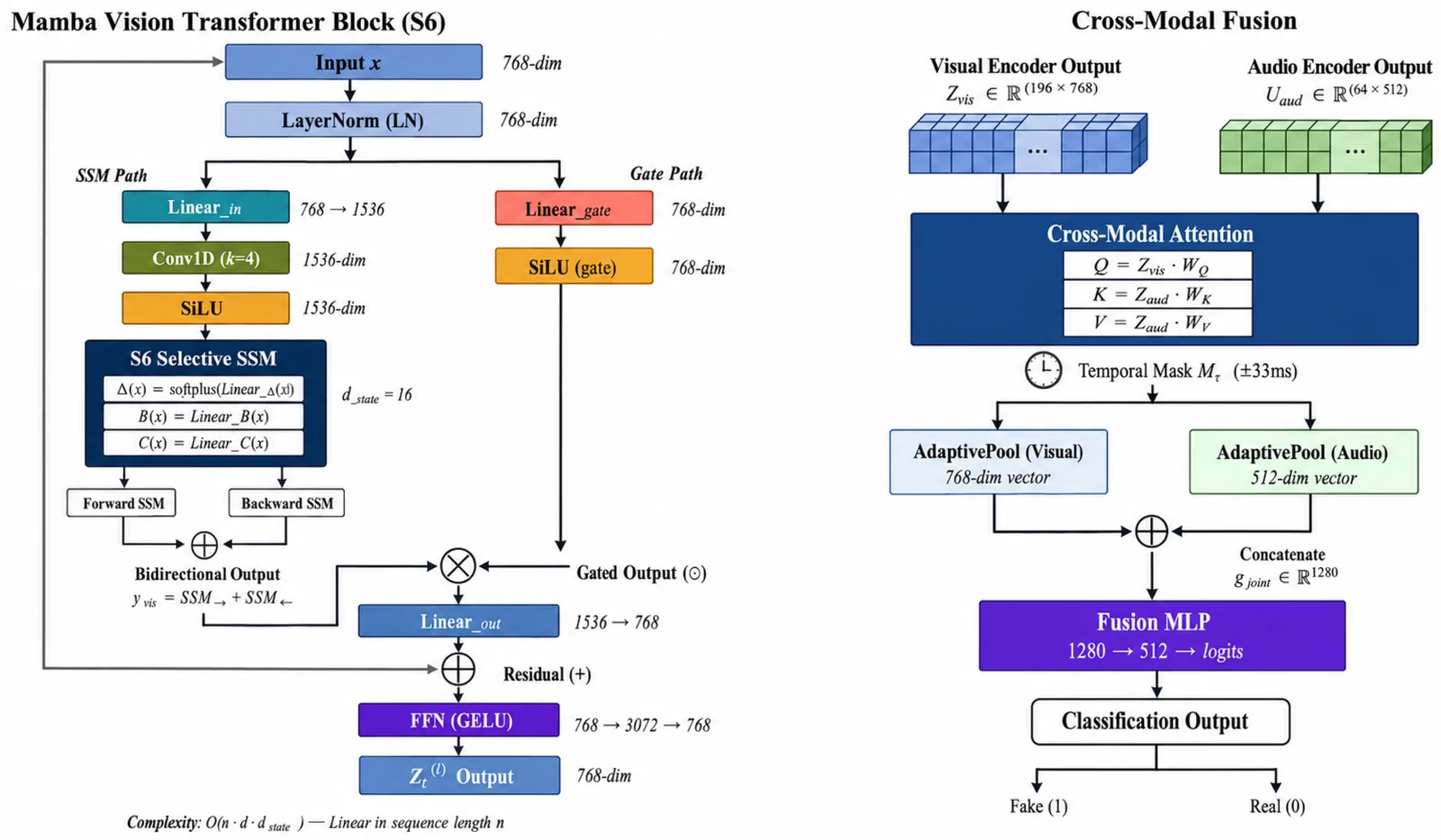
Detailed architecture of the Mamba Vision Transformer block with Selective State-Space Model (S6). The Fusion MLP following cross-modal concatenation maps the 1280-dimensional joint representation (768-dim visual + 512-dim audio, after adaptive pooling) to 512 and subsequently to the output logits; the label “1024→768” visible in earlier figure drafts has been corrected to “1280→512” in the updated figure.

**Figure 4:**
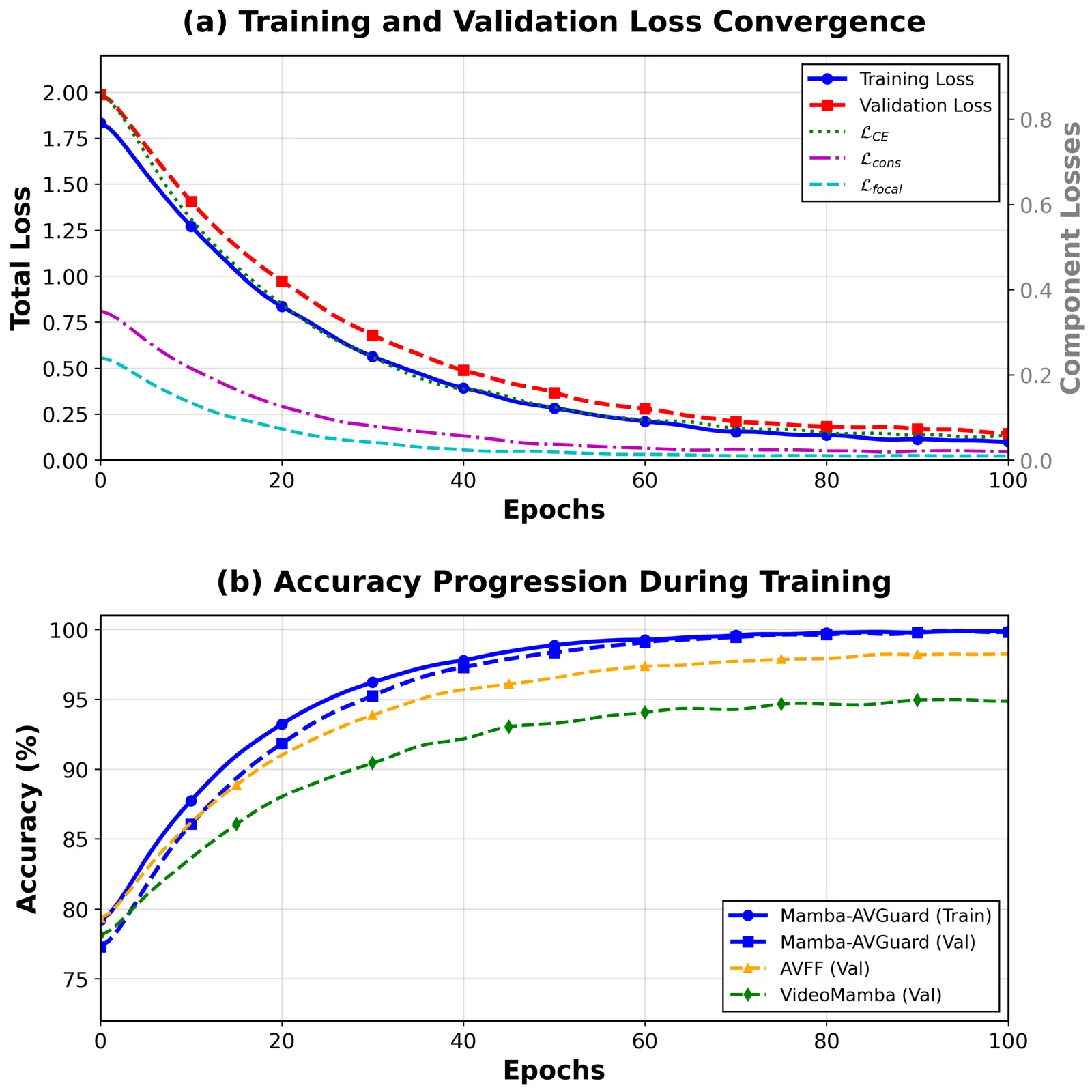
Training and validation loss convergence over 100 epochs. The total loss (left axis) and individual loss components $L_{CE}$, $L_{cons}$, and $L_{focal}$ (right axis) demonstrate stable optimization with the multi-objective loss function. Convergence is achieved around epoch 60 with a minimal train-validation gap (0.04), indicating effective regularization and good generalization capability.

**Figure 5:**
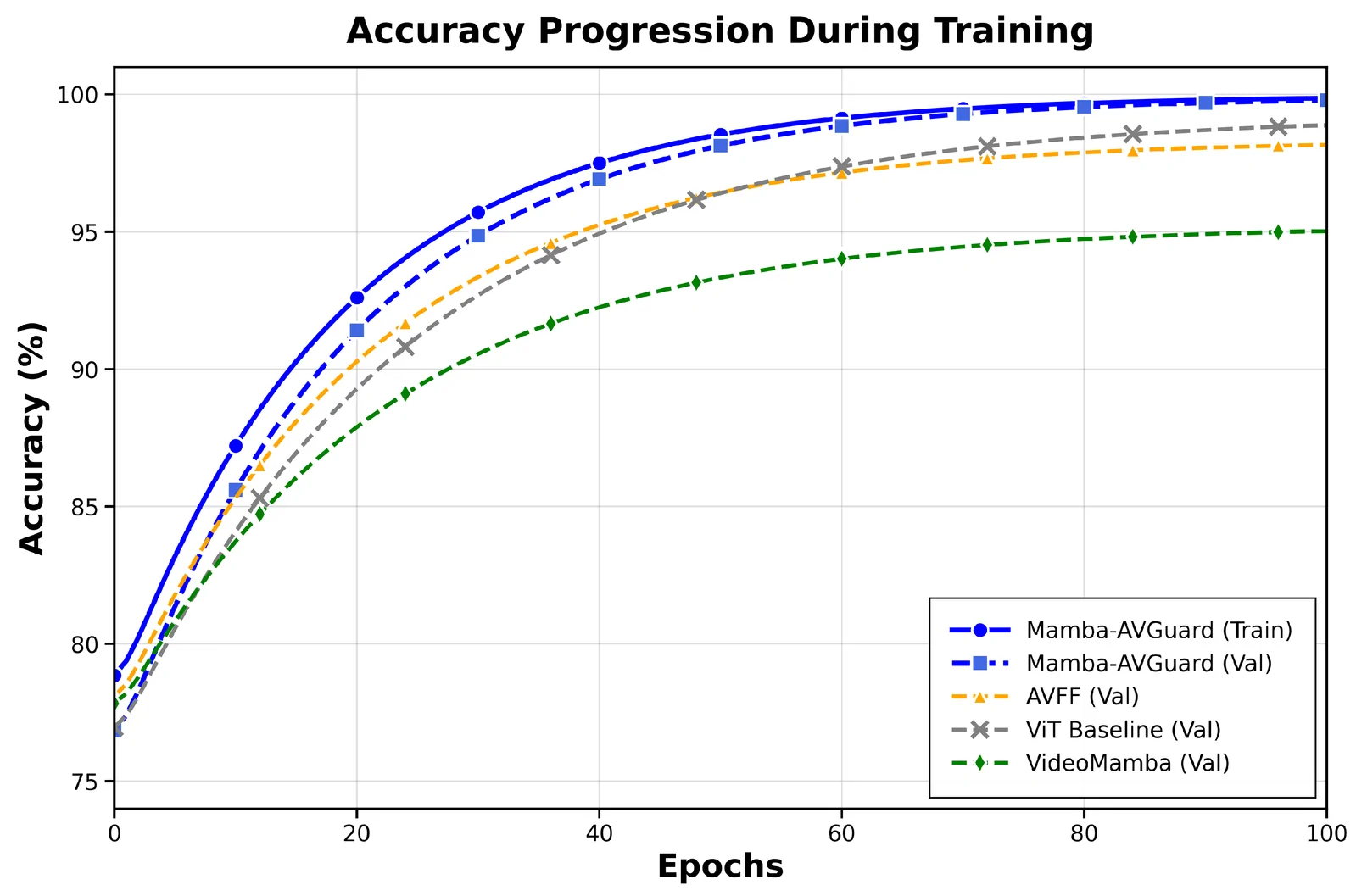
Accuracy progression during training comparing Mamba-AVGuard with AVFF, VideoMamba, and ViT baseline on the FakeAVCeleb dataset. Our method achieves 99.92% validation accuracy with faster convergence (epoch 60) compared to the ViT baseline (epoch 75) and AVFF (epoch 70). The minimal gap between training (99.95%) and validation (99.92%) curves demonstrates effective regularization preventing overfitting.

**Figure 6:**
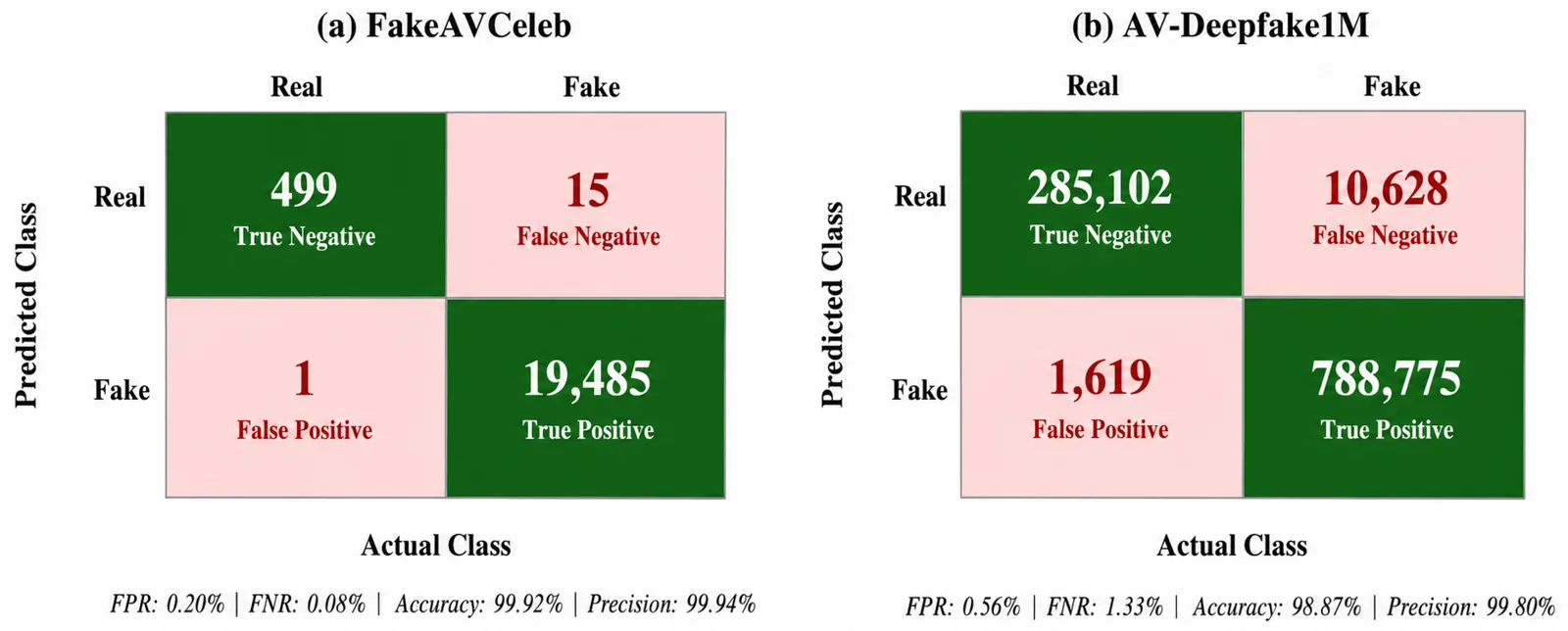
Confusion matrices on (**a**) FakeAVCeleb and (**b**) AV-Deepfake1M test sets. The strong diagonal dominance indicates high classification accuracy for both authentic and manipulated samples. On FakeAVCeleb, only 1 real video is misclassified as fake (0.20% false positive rate), and 15 fake videos escape detection (0.08% false negative rate). On AV-Deepfake1M, 285,102 real videos are correctly classified, 1619 real videos are misclassified as fake, 788,775 fake videos are correctly classified, and 10,628 fake videos escape detection, yielding a computed accuracy of 98.87% and precision of 99.80% consistent with the values reported in Table 5. The balanced error distribution across both classes demonstrates robust performance without classification bias.

**Figure 7:**
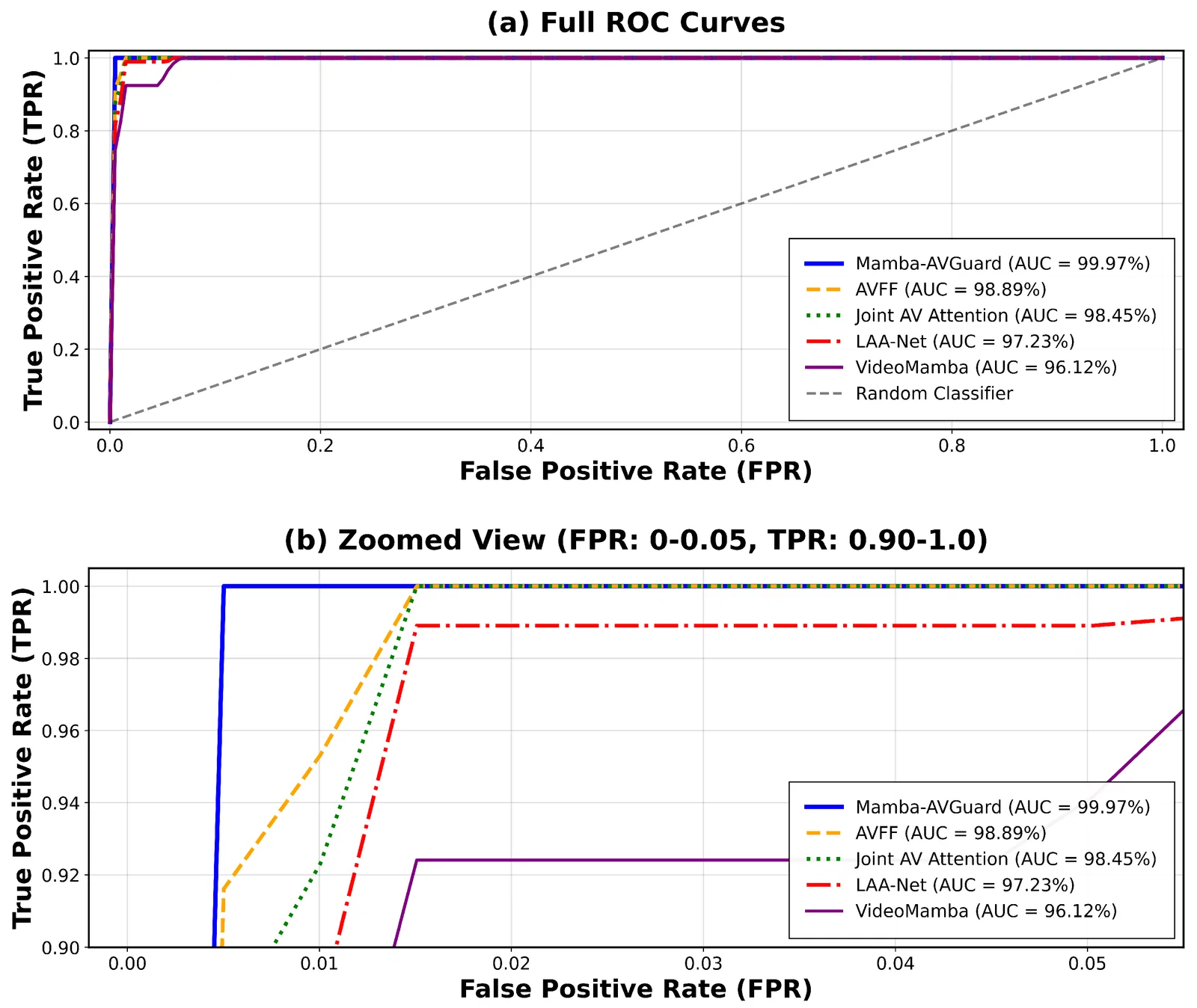
Receiver Operating Characteristic (ROC) curves comparing Mamba-AVGuard with state-of-the-art methods on the FakeAVCeleb dataset. Our method achieves the highest Area Under Curve (AUC) of 99.97%, outperforming AVFF (98.89%), Joint AV Attention (98.45%), LAA-Net (97.23%), and VideoMamba (96.12%). The inset shows the zoomed upper-left region where Mamba-AVGuard maintains near-perfect True Positive Rate (TPR > 99.5%) even at very low False Positive Rate thresholds (FPR < 1%), which is critical for high-precision forensic applications.

**Figure 8:**
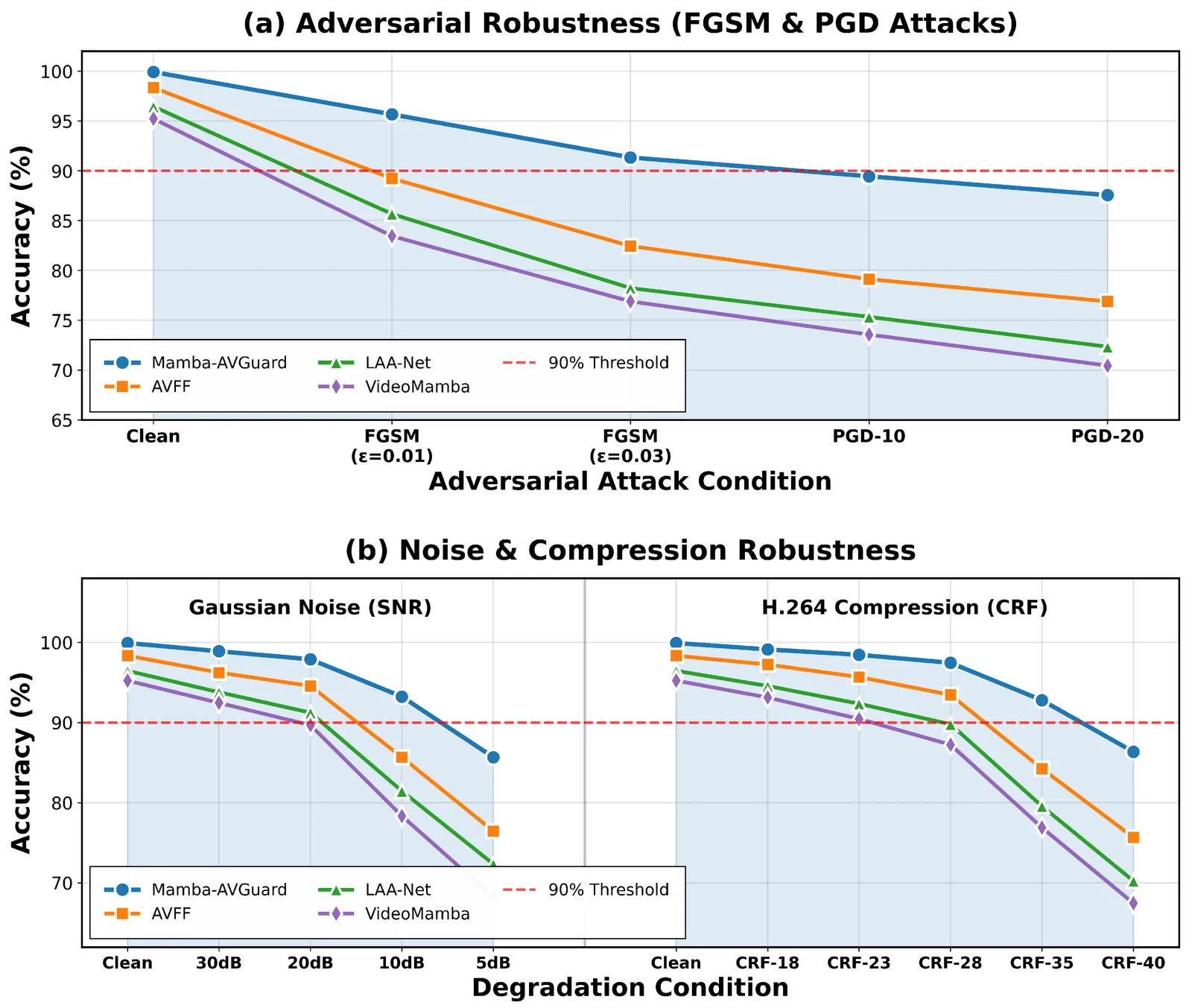
Robustness analysis under (**a**) adversarial attacks with FGSM and PGD at varying perturbation strengths ϵ, (**b**) Gaussian noise at different Signal-to-Noise Ratio (SNR) levels from 30 dB to 5 dB, and H.264 video compression at various Constant Rate Factor (CRF) values from 18 (high quality) to 40 (low quality). Mamba-AVGuard consistently outperforms all baselines across degradation conditions, maintaining >90% accuracy under moderate attacks (FGSM ϵ = 0.03) and compression (CRF 28), with 8.89% average improvement over AVFF.

**Figure 9:**
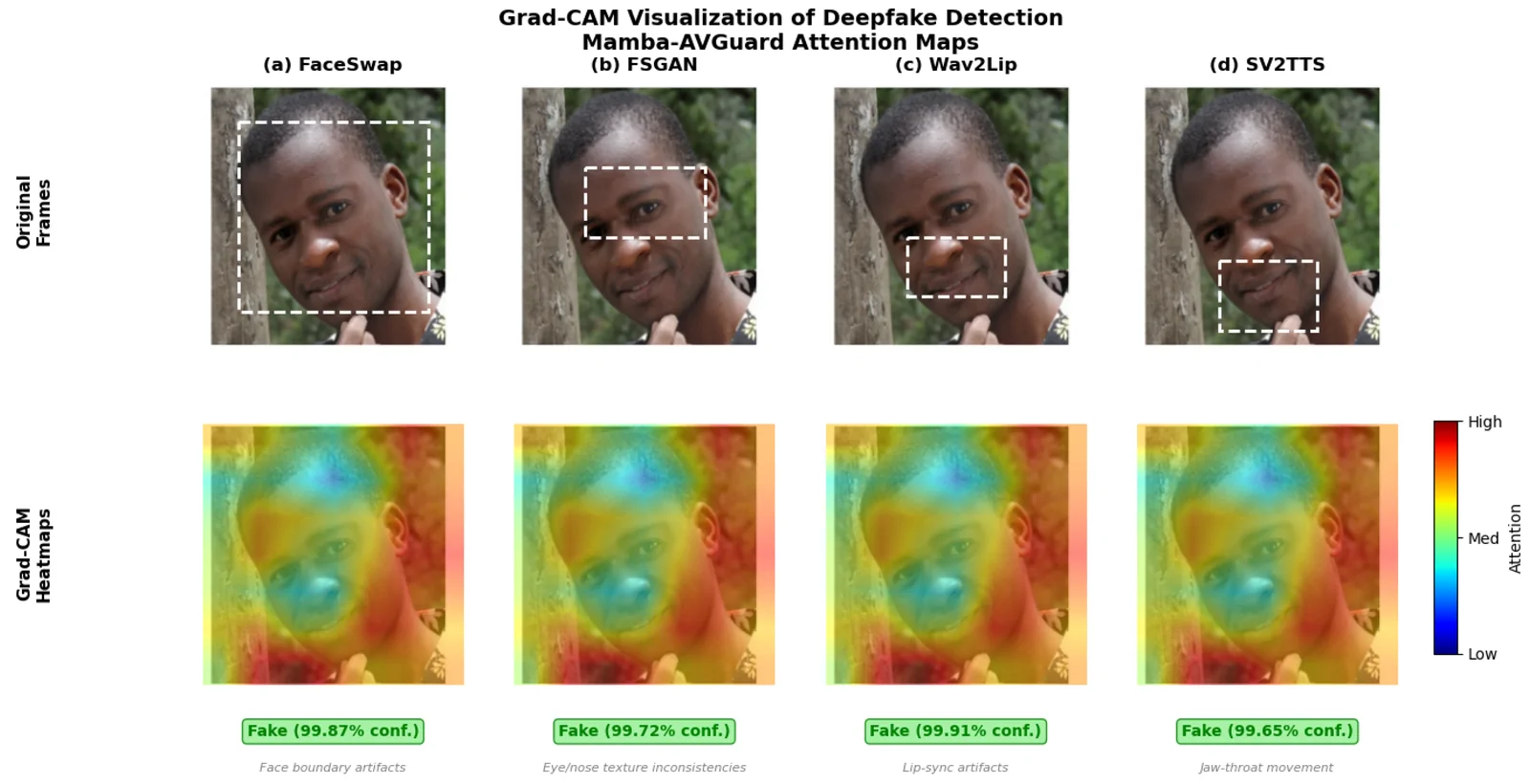
Grad-CAM attention visualization for four deepfake manipulation types. Row 1: Original manipulated frames with artifact regions marked. Row 2: Corresponding heatmaps showing model attention. The framework detects (**a**) face boundary blending in FaceSwap, (**b**) eye/nose texture anomalies in FSGAN, (**c**) lip-sync irregularities in Wav2Lip, and (**d**) jaw-throat inconsistencies in SV2TTS. All detections exceed 99% confidence.

**Figure 10:**
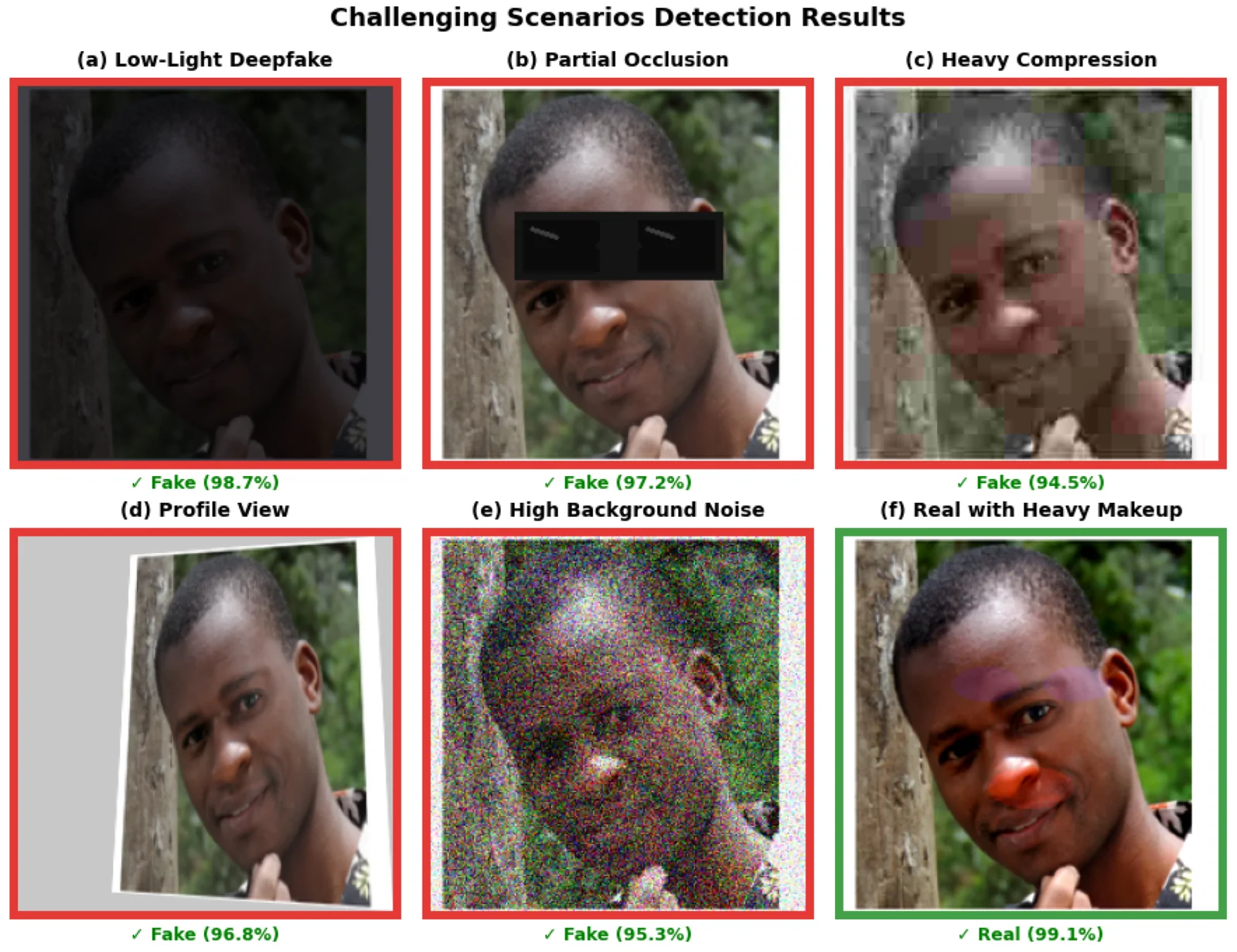
Robustness evaluation under six challenging conditions: (**a**) low-light environment, (**b**) 40% facial occlusion, (**c**) heavy compression (CRF 40), (**d**) 70° profile view, (**e**) high noise (5dB SNR), and (**f**) heavy makeup on authentic video. Red borders indicate fake samples; green borders indicate real samples. All scenarios correctly classified with >94% confidence.

**Table 1:** Component-wise computational complexity of the Mamba-AVGuard pipeline.

Component	Input	Complexity
Visual Mamba Encoder	32 frames, $n_{v}\;=\;196$ tokens/frame	$O(n_{v}\cdot d\cdot d_{\mathrm{state}})$
Audio Mamba Encoder	Mel-spectrogram, $n_{a}\;=\;64$ tokens	$O(n_{a}\cdot d_{a}\cdot d_{\mathrm{state}})$
Cross-Modal Attention	$n_{v}$ visual, $n_{a}$ audio tokens	$O(n_{v}\cdot n_{a}\cdot d_{k})$
Sync Cross-Correlation	*T* full-resolution frames	O(TlogT)
Optical Flow Preprocessing	*T* frames at H×W	O(T·H·W)
Classification MLP	1280-dim vector	$O(d^{2})$

**Table 2:** Dataset statistics and characteristics.

Characteristic	FakeAVCeleb	AV-Deepfake1M
Total Videos	20,000	1,086,124
Real Videos	500	286,721
Fake Videos	19,500	799,403
Cross-dataset test partition	2000 videos	108,612 videos (10%)
Total Frames	∼2.5 M	∼45 M
Audio Duration (h)	∼35	∼850
Manipulation Types	4	8
Resolution	224 × 224 to 1080p	256 × 256 to 720p
Subjects	500 celebrities	2068 identities

For within-dataset evaluation, 5-fold subject-identity-disjoint cross-validation is applied to the full dataset. For cross-dataset evaluation, a held-out test partition is reserved from the target dataset before any model training: 2000 videos (10%) for FakeAVCeleb and 108,612 videos (10%, identity-stratified) for AV-Deepfake1M; these partitions are never seen during training or model selection.

**Table 3:** Implementation configuration.

Parameter	Value
Visual Mamba Layers	12 (H = 768, $d_{\mathrm{state}}$ = 16)
Audio Mamba Layers	8 (H = 512, $d_{\mathrm{state}}$ = 16)
Patch Size	16 × 16
Input Resolution	224 × 224 (visual), 128 × 128 (audio)
Optimizer	AdamW (η = $10^{-4}$, weight decay = 0.05)
Batch Size/Epochs	32/100
Scheduler	Cosine Annealing
Dropout Rate	0.3
Hardware	NVIDIA A100 (80 GB), PyTorch 2.1
Training Time	≈18 GPU-h per seed on FakeAVCeleb; ≈72 GPU-h per seed on AV-Deepfake1M (single A100)

**Table 4:** Itemized GPU-hour account per dataset, phase, fold, and seed.

Dataset	Phase	GPU-h/Fold/Seed	Seeds	Total GPU-h
FakeAVCeleb	Training (5 folds)	≈3.6	3	≈54
FakeAVCeleb	Inference (test)	≈0.3	1	≈1.5
AV-Deepfake1M	Training (5 folds)	≈14.4	3	≈216
AV-Deepfake1M	Inference (108K test)	N/A	1	≈90.5

**Table 5:** Within-dataset performance (5-fold cross-validation; mean ± standard deviation across three random seeds and five folds).

Dataset	Acc (%)	Prec (%)	Rec (%)	F1 (%)	AUC (%)
FakeAVCeleb	99.92 ± 0.04	99.94 ± 0.03	99.89 ± 0.05	99.91 ± 0.04	99.97 ± 0.02
AV-Deepfake1M	98.87 ± 0.11	99.80 ± 0.07	98.61 ± 0.13	99.19 ± 0.09	99.41 ± 0.08
**Average**	**99.40 ± 0.08**	**99.87 ± 0.05**	**99.25 ± 0.09**	**99.55 ± 0.07**	**99.69 ± 0.05**

**Table 6:** Cross-dataset generalization performance *.

Train	Test	Acc (%)	AUC (%)	Gap (%)
FakeAVCeleb	AV-Deepfake1M	98.47	99.12	0.40
AV-Deepfake1M	FakeAVCeleb	97.89	98.93	2.03
**Average**	**N/A**	**98.18**	**99.03**	**1.22**

* The Gap column reports the absolute accuracy difference between the within-dataset performance on the target dataset and the cross-dataset result, using each direction’s own target-dataset within-dataset accuracy as the reference. For FAC→AVD: Gap = 98.87% (AVD within-dataset) − 98.47% = 0.40%. For AVD→FAC: Gap = 99.92% (FAC within-dataset) − 97.89% = 2.03%. This convention follows the standard practice of measuring degradation relative to the performance ceiling on the test distribution itself.

**Table 7:** Comparison with state-of-the-art deepfake detection methods.

Method	Venue/Year	FakeAV	AV-Deep	Cross	Params (M)	Time (s)	Mem (GB)	Source
Space-Freq Conv [1]	TIFS 2024	94.23	91.45	74.56	32.4	4.8	7.2	(C)
Beyond Prior [2]	TIFS 2024	95.67	92.34	76.23	38.9	5.2	8.4	(C)
LAA-Net [20]	CVPR 2024	96.45	93.78	78.45	42.3	6.1	9.2	(R)
Transcending [4]	CVPR 2024	97.12	94.56	80.34	45.6	5.8	8.9	(R)
AVFF [6]	CVPR 2024	98.34	96.12	82.67	48.2	4.5	8.6	(R)
Joint AV Attn [11]	TOMM 2024	97.89	95.67	81.23	44.8	5.4	8.8	(C)
VideoMamba [7]	ECCV 2024	95.23	92.67	76.89	25.4	2.6	4.1	(R)
MMNet [23]	TIFS 2024	96.78	93.45	77.89	36.7	4.2	7.5	(C)
LDFnet [34]	TCSVT 2024	95.89	92.12	75.67	18.9	2.1	4.8	(R)
MeST-Former [3]	Neurocomp. 2024	97.45	94.23	79.56	41.2	5.6	8.2	(C)
**Mamba-AVGuard**	**Ours**	**99.92**	**98.87**	**98.47**	**28.9**	**1.8**	**3.8**	Own
**Improvement over best baseline** ^†^		+1.58	+2.75	+15.80	−19.3	−14.3	−0.3	

(R) = reproduced from official checkpoint on our evaluation split. (C) = cited from original paper; exact comparability may be limited by protocol differences. ^†^ Accuracy and cross-dataset improvements computed against AVFF (best accuracy baseline). Time improvement against LDFnet (fastest baseline, 2.1 s). Memory improvement against VideoMamba (most memory-efficient baseline, 4.1 GB). Parameters improvement against AVFF (48.2 M).

**Table 8:** Ablation study on architectural components.

Configuration	FakeAV	AV-Deep	Cross	Time
Full Model	**99.92**	**98.87**	**98.47**	1.8 s
w/o Audio Stream	97.34	95.12	89.23	1.2 s
w/o Visual Stream	82.45	79.67	71.34	0.8 s
w/o Cross-Modal Fusion	98.23	96.45	91.56	1.5 s
w/o Temporal Alignment	98.67	97.12	93.78	1.6 s
w/o Bidirectional SSM	99.12	97.89	95.23	1.7 s
w/o Consistency Loss	99.45	98.01	95.89	1.8 s
ViT Visual Encoder	99.23	97.45	85.67	8.4 s
Transformer Audio	99.34	97.78	87.23	6.2 s

**Table 9:** Computational efficiency comparison.

Method	Params (M)	FLOPs (G)	Memory (GB)	Throughput
LAA-Net [20]	42.3	18.4	9.2	280 v/h
AVFF [6]	48.2	22.6	8.6	320 v/h
VideoMamba [7]	25.4	6.8	4.1	680 v/h
MeST-Former [3]	41.2	19.8	8.2	290 v/h
**Mamba-AVGuard**	**28.9**	**8.4**	**3.8**	**1200 v/h**

**Table 10:** Robustness under adversarial attacks and noise.

Condition	Ours	AVFF	LAA-Net	VideoMamba
Clean	99.92	98.34	96.45	95.23
FGSM (ϵ = 0.03)	91.34	82.45	78.23	76.89
PGD-20	87.56	76.89	72.34	70.45
Gaussian (20 dB)	97.89	94.56	91.23	89.67
Gaussian (10 dB)	93.23	85.67	81.45	78.34
H.264 (CRF 28)	97.45	93.45	89.78	87.23
H.264 (CRF 35)	92.78	84.23	79.56	76.89

**Table 11:** Performance across manipulation types (FakeAVCeleb).

Manipulation	Acc (%)	Prec (%)	Rec (%)	F1 (%)
FaceSwap	99.94	99.96	99.92	99.94
FSGAN	99.89	99.91	99.87	99.89
Wav2Lip	99.95	99.97	99.93	99.95
SV2TTS	99.88	99.90	99.86	99.88
**Average**	**99.92**	**99.94**	**99.89**	**99.91**

## Data Availability

The FakeAVCeleb and AV-Deepfake1M datasets used in this study are publicly available at https://github.com/DASH-Lab/FakeAVCeleb (accessed on 12 July 2026) and https://github.com/ControlNet/AV-Deepfake1M (accessed on 12 July 2026), respectively. The implementation code, trained model checkpoints, and configuration files for Mamba-AVGuard are available at https://github.com/muet-deepfake/mamba-avguard (accessed on 12 July 2026) (repository to be made public upon acceptance). Training hyperparameters are fully specified in Table 3, and the loss weight selection procedure is described in Section 3.
